# Neighborhood deprivation and lung cancer incidence and mortality in patients with asthma: a nationwide cohort study in Sweden, 1997–2018

**DOI:** 10.1186/s12931-026-03927-5

**Published:** 2026-09-22

**Authors:** Juan Cao, Tsuyoshi Hamano, Huifang Yang, Filip Jansåker, Kristina Sundquist, Jan Sundquist, Yuhong Zhang, Xinjun Li

**Affiliations:** 1 Department of Childhood Health and Health Education, School of Public Health, Ningxia Medical University, Ningxia, China; 2 Key Laboratory of Environmental Factors and Chronic Disease Control, Ningxia, China; 3 Center for Primary Health Care Research, Department of Clinical Sciences Malmö, Lund University, Jan Waldenströms gata 35, Malmö, 20502 Sweden; 4 University Clinic Primary Care, Skåne University Hospital, Region Skåne, Sweden; 5 Department of Sports Sociology and Health Sciences, Kyoto Sangyo University, Kyoto, Japan; 6 Center for Community-Based Healthcare Research and Education (CoHRE), Head Office for Research and Academic Information, Shimane University, Izumo, Japan; 7 Departments of Family and Community Medicine and of Epidemiology, The University of Texas Health Science Center, Houston, TX USA

**Keywords:** Lung cancer, Asthma, Neighborhood deprivation, Mortality

## Abstract

**Objective:**

This population-based study aimed to investigate the association between neighborhood deprivation and the incidence and mortality of lung cancer in patients with asthma.

**Methods:**

This nationwide cohort study included 596,215 individuals aged ≥ 25 years with asthma who were followed from 1997 to 2018 in Sweden. Cox proportional hazards regression was used to estimate hazard ratios (*HRs*) and 95% confidence intervals (*CI*s) for lung cancer incidence and mortality associated with neighborhood deprivation. All models were stratified by sex and adjusted for individual-level characteristics, including age, education level, sociodemographic factors, and comorbidities.

**Results:**

Neighborhood deprivation was independently associated with both lung cancer incidence and mortality in patients with asthma, even after adjustment for individual-level characteristics. In fully adjusted models, compared with individuals living in low-deprivation neighborhoods, those in high-deprivation neighborhoods had a significantly elevated risk of lung cancer incidence (*HR* = 1.37) and mortality (*HR* = 1.40), with more pronounced associations observed in women. Histological subtype analyses revealed differential patterns: high deprivation was associated with increased risks of adenocarcinoma (*HR* = 1.19) and large cell carcinoma (*HR* = 1.58), whereas squamous cell carcinoma (*HR* = 1.75) and small cell carcinoma (*HR* = 1.65) showed increased risks in both moderate- and high-deprivation neighborhoods.

**Conclusion:**

Neighborhood deprivation may be a critical social determinant of lung cancer incidence and mortality among patients with asthma, with effects varying by age, sex, and histological subtype. Targeted interventions in deprived communities could consider prioritizing high-risk groups, individuals with low educational attainment, and those with comorbid chronic obstructive pulmonary disease.

**Supplementary Information:**

The online version contains supplementary material available at https://doi.org/10.1186/s12931-026-03927-5.

## Introduction

Globally, the age-standardized incidence rate of asthma decreased by 13% between 1990 and 2019, from 580.1 to 504.3 per 100,000 population [1]. Despite this decline, asthma remains a highly prevalent chronic respiratory disorder [2, 3] and the total number of people with asthma is increasing in many countries primarily driven by population growth. Extensive epidemiological research has established associations between asthma and comorbidities such as metabolic syndrome, chronic obstructive pulmonary disease (COPD), and cardiovascular diseases [4, 5]. Asthma has also been linked to several malignancies, including breast cancer and stomach cancer, but its relationship with lung cancer remains inconsistent [6]. Many studies have suggested that asthma may increase lung cancer risk [7–10], whereas some research has found no significant association [11]. The observed discrepancies may be attributable to differences in confounder adjustment, thus emphasizing the need for rigorous investigations into potential mechanisms linked to lung cancer in patients with asthma. Such studies are important given that lung cancer is the leading cause of cancer-related mortality worldwide (18.0% of total cancer deaths) and is the second most frequently diagnosed cancer (11.4% of all cases) [12]. Furthermore, lung cancer directly affects respiratory health and imposes substantial healthcare and economic burdens on society [7, 10, 13].

Neighborhood deprivation, a multidimensional measure of socioeconomic disadvantage, has been independently linked to both asthma severity and lung cancer outcomes [7, 14–16]. For instance, children living in highly deprived neighborhoods exhibit higher asthma prevalence, more severe symptoms, and greater emergency department utilization than those residing in more affluent areas [17, 18]. Similarly, neighborhood deprivation has been linked to increased lung cancer incidence and mortality in the general population [19]. Nevertheless, whether this environmental stressor influences lung cancer risk or prognosis specifically among individuals with asthma remains unknown. Addressing this knowledge gap could enhance risk stratification and inform novel prevention strategies for this vulnerable subgroup. To date, no studies have examined the relationship between neighborhood deprivation and lung cancer risk in patients with asthma.

Clarifying this relationship holds promise for developing targeted prevention strategies and improving survival outcomes for the growing asthma population globally. Despite the high global prevalence of asthma and the poor survival rates associated with lung cancer, existing research has largely focused on individual-level socioeconomic factors, with limited attention to community-level determinants. Moreover, longitudinal cohort studies that integrate multidimensional social and environmental variables remain scarce. It is therefore critical to determine whether neighborhood deprivation constitutes a risk factor for lung cancer among patients with asthma.

To address this gap, we propose a nationwide retrospective cohort study designed to systematically evaluate the association between neighborhood deprivation and lung cancer incidence and mortality in patients with asthma. The findings are expected to inform precision public health interventions aimed at reducing cancer disparities among this high-risk population.

## Methods

### Study design and setting

This population-based nationwide cohort study used individual-level data derived from comprehensive national registers and primary healthcare records covering all residents of Sweden. The study population was comprised of individuals aged ≥ 25 years with a diagnosis of asthma. The index date (baseline) was defined as the date when an individual met the age and asthma criteria. The study was conducted at the Center for Primary Healthcare Research, Lund University, Sweden.

### Data sources

Data were derived from comprehensive national registers and primary care data containing individual-level information for all residents, including age, sex, socioeconomic status, occupation, residential region, hospital diagnoses, admission dates, emigration dates, and causes of death. Record linkage was performed using Sweden’s unique 10-digit personal identifier, which is assigned at birth or immigration. The following registers were used: Swedish Cancer Register (1958–2018), National Patient Register (Inpatient data, 1964–2018 and Outpatient data, 2001–2018); Cause of Death Register (1961–2018); Total Population Register (1968–2018), and Primary Health Care Register (1990–2018).

### Study population

We identified 1,152,825 unique patients with asthma diagnosed during 1990 to 2018, according to the 9th and 10th editions of the International Classification of Diseases: ICD–9 codes 493 from 1990 to 1996, and ICD-10 codes J45–46 from 1997 to 2018. After excluding those aged < 25 years, individuals with unknown neighborhood deprivation information, and those with preexisting lung cancer (diagnosis), a total of 596,215 patients with asthma remained suitable for inclusion in the study population (Figure S1).

### Ascertainment of outcome variables

The Swedish Cancer Register (ICD–7 162, 163) and Cause of Death Register (ICD–10 C33, C34) were used to identify the outcome variables of lung cancer incidence and lung cancer mortality (cause-specific for lung cancer) during the study period (1997–2018). Lung cancer incidence was identified in the Swedish Cancer Registry (established in 1958). In this register, all cancer diagnoses for the coding site have been translated to comply with ICD–7 [20]. Lung cancer mortality was identified in the Swedish Cause of Death Register (established in 1961) as either primary or secondary cause of death [21]. Patients with a lung cancer diagnosis registered before baseline were excluded to ensure that all incident lung cancers were the first registered diagnosis in each individual.

### Individual variables

All individual-level sociodemographic variables were assessed from the Swedish Total Population Register at the beginning of follow-up for each individual (baseline). Sex was categorized into male or female. *Age* was categorized as 25–34, 35–44, 45–54, 55–64, 65–74, and ≥ 75 years of age. *Educational level* was divided into three groups based on: completion of compulsory school or less (≤ 9 years: low), practical high school or some theoretical high school (10–12 years: middle), or theoretical high school and/or college (> 12 years: high). *Marital status* was divided into two groups: married/cohabitating, and never married, widowed, or divorced. *Family income* was categorized into four groups (Low, including individuals in the lowest family income quartile; Middle low, including individuals in the middle low quartile; Middle high, including individuals in the middle high quartile; High, including individuals in the highest quartile) based on the annual family income divided by the number of people in the family, i.e., individual family income per capita. *Country of origin* was divided into two groups: born in Sweden and born outside of Sweden. *Region of residence* was divided into three groups: large cities (Stockholm, Göteborg, Malmö), southern Sweden, and northern Sweden. *Comorbidities* were identified from national patient registers during the study period: chronic obstructive pulmonary disease (COPD) (J44), alcoholism (F10), and clinically diagnosed smoking (Z72.0), below referred to as “smoking”.

### Neighborhood-level variable

Neighborhood Deprivation Index is a summary measure that has previously been used to characterize neighborhood-level deprivation [22, 23]. The home addresses of all Swedish adults have been geocoded to small geographic units that have boundaries defined by similar types of buildings (Figure S2). These neighborhood areas are called small area market statistics (SAMS) and have an average of 1000 to 2000 residents and were used as proxies for neighborhoods, as has been done in previous research [22]. The four variables were selected based on the established neighborhood deprivation index developed by Winkleby et al. [23], allowing comparability with previous Swedish studies. The following four variables were selected: low educational status (below 10 years of formal education); Low income (income from all sources, including that from interest and dividends, defined as less than 50% of individual median income) [24]; Unemployment (not employed, excluding full-time students, those completing compulsory military service, and early retirees); and social welfare assistance.

Each of the four variables loaded on the first principal component with coefficients ranging from + 0.47 to + 0.53, collectively explaining 52% of the inter-variable variance. A standardized z-score was computed for each SAMS neighborhood [22]. These z-scores were then weighted by their respective eigenvector coefficients and summed to construct the deprivation index [25], where higher values indicate greater neighborhood deprivation. The index was categorized into three levels based on standard deviation (SD) from the mean: low deprivation (> 1 SD below mean), moderate deprivation (within ± 1 SD of mean), and high deprivation (> 1 SD above mean). Residents of low-deprivation neighborhoods (representing the most affluent areas) were defined as the reference group. Deprivation was assessed cross-sectionally at baseline (Table S1).

### Statistical analysis

Descriptive statistics and rates of first lung cancer incidence/mortality events were calculated for the overall study population, stratified by neighborhood deprivation level, age, and sex. Person-years were computed from the start of follow-up (baseline) until each individual’s first diagnosis of lung cancer/mortality of lung cancer, death, emigration, or the end of the study on December 31, 2018. Sex-stratified Cox proportional hazards models estimated hazard ratios (*HR*s) with 95% confidence intervals (*CI*s) for lung cancer incidence and mortality in relation to neighborhood deprivation, adjusting for individual characteristics and comorbidities. The first model in the analysis was a univariate Cox regression performed for each variable. Secondly, stepwise multivariate Cox regression included the primary predictor and covariates. A full model without stratification by sex was also conducted. Further adjustment was made for competing risks for mortality due to cardiovascular diseases as a competing risk for lung cancer mortality. The interrelationships between the variables that may affect the exposure and outcome variables were plotted in a directed acyclic graph (DAG) (Figure S3) using the software at Dagitty.net (version 3.0) [26] (see Table S2 for Digital Content for the full code for reproducing our DAG at Dagitty.net). The variables’ correlation with each other, the exposure, and the outcome measure were considered and marked with a pointed arrow in the potentially affected direction, as found relevant from a clinical standpoint and the medical literature.

A sensitivity analysis was performed to examine the association between neighborhood deprivation and lung cancer in patients with asthma after excluding lung cancer diagnosed before asthma, so as to identify the robustness and reliability of the models.

As an additional sensitivity analysis, individuals with a previous diagnosis of lung cancer (1990–1996) were excluded. To assess potential temporal variation in the association between neighborhood deprivation and lung cancer outcomes, an additional sensitivity analysis was performed by stratifying patients according to the period of asthma diagnosis (1997–2007 and 2008–2018).

As another sensitivity analysis, neighborhood deprivation was additionally modeled as a continuous variable (per one-unit increase) rather than a categorical variable to assess the robustness of the results and to examine whether the association with lung cancer risk was consistent across the full deprivation scale.

To assess the potential influence of individual socioeconomic status on the observed associations, an additional sensitivity analysis was performed by stratifying individuals according to their educational level. The association between neighborhood deprivation and lung cancer outcomes was examined separately within each educational category.

All analyses were performed using SAS 9.4 (SAS Institute Inc., Cary, NC, USA).

## Results

A total of 596,215 individuals aged ≥ 25 years with asthma and without preexisting lung cancer were included in the study. Neighborhoods were categorized into low (25.7%), moderate (56.8%), and high (17.5%) deprivation levels (Table S1).

Among patients with asthma, 5,473 lung cancer cases and 4,439 deaths due to lung cancer were recorded, peaking in ages 65–74 (24.7%/34.6% of incidence/deaths). Despite females comprising 60.5% of the cohort, they had higher incidence (63.2%) and mortality (62.3%). Rates per 1,000 rose with deprivation (incidence: low = 7.2, high = 10.8). Low education was correlated with sharply elevated incidence (15.6 vs. 5.1/1,000 in higher education). COPD showed the strongest risk association (rates = 10.7–17.1/1,000), underscoring deprivation (Table 1, Table S3).

**Table 1:** Population of lung cancer incidence and mortality due to lung cancer among patients with asthma

Variables	Total Population		Lung cancer incidence		Neighborhood deprivation level-specific rate per 1,000			Mortality due to lung cancer		Rate per 1000 in neighborhood deprivation level		
	No.	%	No.	%	Low	Moderate	High	No.	%	Low	Moderate	High
Total population	596,215		5473		1028	3175	1270	4439		773	2533	1133
Standardized overall rate*(per 1000 person years)			5.8		4.7	5.5	7.6	4.3		3.4	4.1	6.5
Total cumulative rate per 1000 individuals					7.2	9.5	10.8			5.4	7.6	9.7
Age (years)												
25–34	96,077	16.1	38	0.7	0.2	0.6	0.2	15	0.3	0.0	0.3	0.0
35–44	109,268	18.3	209	3.8	1.3	2.2	2.0	109	2.5	0.5	1.2	1.0
45–54	105,235	17.7	728	13.3	4.3	7.5	8.8	556	12.5	3.1	5.4	8.0
55–64	103,619	17.4	1633	29.8	12.3	15.8	20.1	1155	26.0	8.2	11.3	14.8
65–74	94,191	15.8	1853	33.9	17.2	19.1	24.7	1535	34.6	13.1	15.5	22.7
≥ 75	87,825	14.7	1012	18.5	11.7	10.6	14.0	1069	24.1	11.5	11.1	15.9
Sex												
Males	235,734	39.5	2012	36.8	6.6	8.6	10.6	1673	37.7	5.1	7.1	9.7
Females	360,481	60.5	3461	63.2	7.5	10.0	11.0	2766	62.3	5.6	7.9	9.7

* Adjusted for the European standardized population

**Fig. 1:**
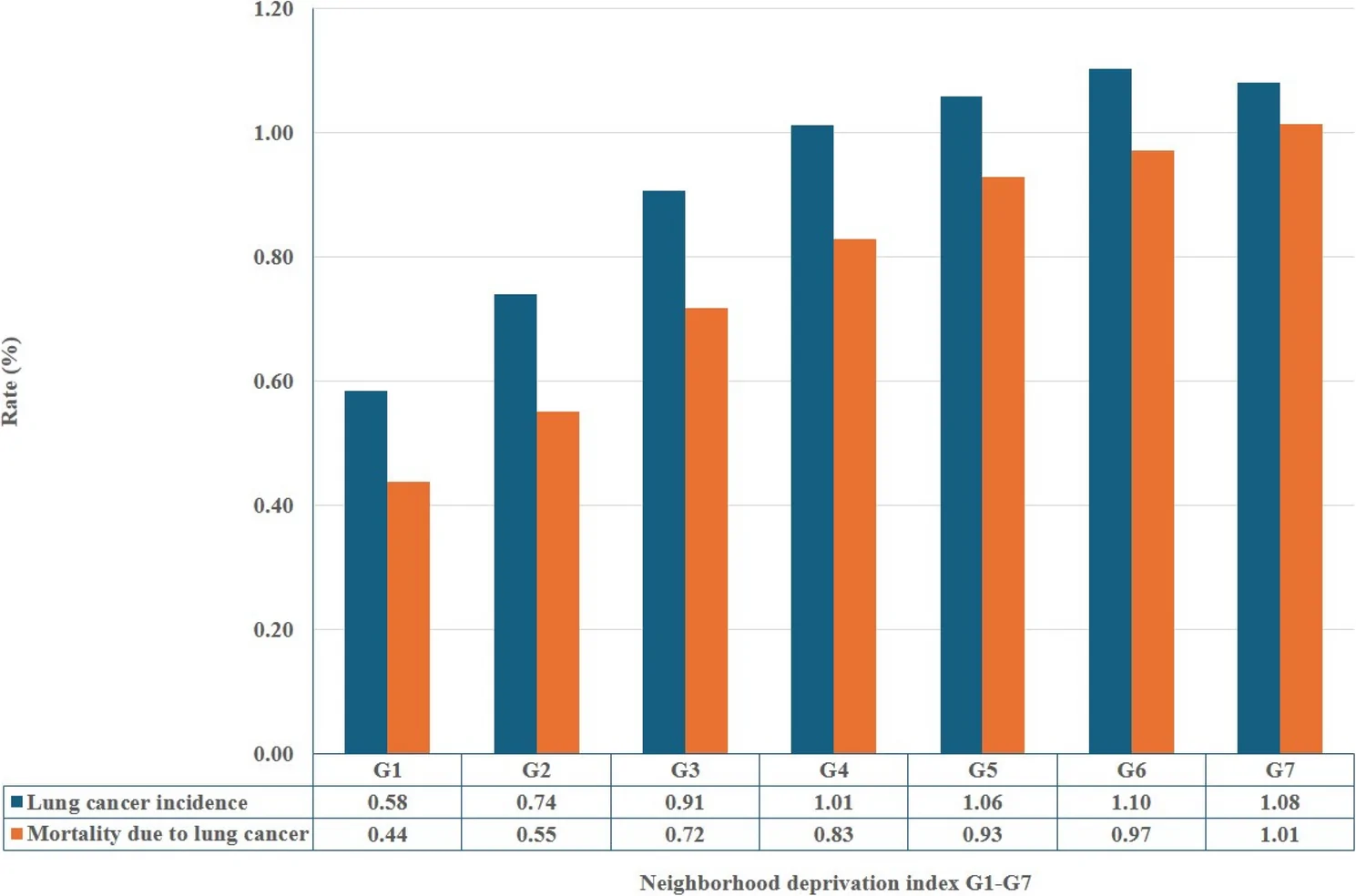
Cumulative rate (%) of lung cancer and mortality due to lung cancer among patients with asthma by neighborhood deprivation index: G1 (Richest) -3.0 to -2.0, G2 -1.9 to -1.0, G3 -0.9 to 0.0, G4 0.0 to 0.9, G5 1.0 to 1.9, G6 2.0 to 2.9, G7 (poorest) 3.0 to 10.5

Figure 1 shows the cumulative incidence rate of lung cancer and mortality due to lung cancer by neighborhood deprivation in the total study population. The rate of patients diagnosed with lung cancer and mortality due to lung cancer among patients with asthma increased when neighborhood deprivation increased.

Table 2 demonstrated the association between neighborhood deprivation and lung cancer incidence among patients with asthma. The results showed that a high-deprivation neighborhood was strongly associated with increased risk of lung cancer incidence, with unadjusted *HR* = 1.52 (95% *CI*: 1.40–1.65) and adjusted *HR* = 1.35. Men in high-deprivation areas faced elevated risks even after adjusting for demographics, socioeconomic factors, and comorbidities, compared to women. Smoking (*HR* = 3.09, 95% *CI*: 2.74–3.50) and COPD (*HR* = 2.26, 95% *CI*: 2.13–2.39) were dominant risk factors, particularly in women. Age amplified risks in both sexes, whereas marital and immigrant status had non-significant impact (Table S4, S5, S6).

**Table 2:** Association between neighborhood deprivation and lung cancer incidence among patients with asthma stratified by sex

Variables	Model 1		Model 2		Model 3	
	HR	95% CI	HR	95% CI	HR	95% CI
Men						
Neighborhood deprivation (ref. Low)						
Moderate	1.09	0.97–1.22	1.04	0.92–1.17	1.05	0.93–1.18
High	1.61	1.41–1.84	1.36	1.19–1.57	1.37	1.19–1.57
Women						
Neighborhood deprivation (ref. Low)						
Moderate	1.18	1.08–1.29	1.12	1.02–1.22	1.12	1.02–1.22
High	1.47	1.33–1.64	1.34	1.20–1.49	1.32	1.18–1.47
Both sexes*						
Neighborhood deprivation (ref. Low)						
Moderate	1.15	1.07–1.23	1.09	1.02–1.17	1.10	1.02–1.18
High	1.52	1.40–1.65	1.36	1.25–1.48	1.35	1.24–1.47

Model 1: Adjusted for age; Model 2: Adjusted for age, educational level, marital status, immigrant status, and region of residence; Model 3: Adjusted for age, educational level, marital status, immigrant status, region of residence, and comorbidities

*HR* Hazard ratio, *CI* Confidence interval

* Sex was adjusted in model 2 and model 3

Neighborhood deprivation was significantly associated with higher lung cancer mortality in patients with asthma, with sex-specific patterns. For men, high-deprivation neighborhoods showed elevated risks (*HR* = 1.64, 95%*CI*: 1.55–1.74), persisting post-adjustment (*HR* = 1.40, 95%*CI*: 1.32–1.49). Similarly, women exhibited higher mortality in high-deprivation neighborhoods (*HR* = 1.43, 95%*CI*: 1.38–1.49) (Table 3).

**Table 3:** Association between neighborhood deprivation and mortality due to lung cancer among patients with asthma stratified by sex

Variables	Model 1		Model 2		Model 3	
	HR	95% CI	HR	95% CI	HR	95% CI
Men						
Neighborhood deprivation (ref. Low)						
Moderate	1.23	1.17–1.29	1.08	1.02–1.13	1.10	1.04–1.15
High	1.64	1.55–1.74	1.38	1.30–1.47	1.40	1.32–1.49
Women						
Neighborhood deprivation (ref. Low)						
Moderate	1.26	1.21–1.32	1.14	1.09–1.19	1.15	1.10–1.20
High	1.66	1.58–1.74	1.44	1.38–1.52	1.46	1.39–1.54
Both sexes*						
Neighborhood deprivation (ref. Low)						
Moderate	1.25	1.21–1.29	1.11	1.07–1.15	1.13	1.09–1.16
High	1.64	1.58–1.71	1.41	1.36–1.47	1.43	1.38–1.49

Model 1: Adjusted for age; Model 2: Adjusted for age, educational level, marital status, immigrant status, and region of residence; Model 3: Adjusted for age, educational level, marital status, immigrant status, region of residence, and comorbidities

*HR* Hazard ratio, *CI* Confidence interval

* Sex was adjusted in model 2 and model 3

The analysis across Tables S7 (males), S8 (females), and S9 (general population) highlights that higher neighborhood deprivation strongly correlates with elevated lung cancer mortality in patients with asthma, with adjusted risks for high deprivation in males and in females. Age increased mortality annually, while low education and unmarried status further exacerbated risks. Comorbidities such as COPD (*HR* = 1.67–1.70), alcoholism (*HR* = 1.68–1.91), and smoking (*HR* = 1.16–1.40) were associated with higher mortality. Sex disparities emerged: males had higher overall mortality (*HR* = 1.41 vs. females), while females exhibited geographic (Northern Sweden: *HR* = 1.07) and differences in marital status-being never married, widowed, or divorced were associated with higher mortality (*HR* = 1.11).

We further demonstrated age-stratified associations between neighborhood deprivation and lung cancer risk using fully adjusted models among 5,473 patients with both lung cancer and asthma. High deprivation was consistently associated with elevated risk across age groups: < 55 years (*HR* = 1.43, 95%*CI*: 1.16–1.77), 55–64 years (*HR* = 1.44, 95%*CI*: 1.23–1.69), and ≥ 75 years (*HR* = 1.39, 95%*CI*: 1.15–1.69). Moderate deprivation was associated with higher risk only in younger patients (< 55 years: *HR* = 1.37, 95%*CI*: 1.14–1.63), while showing no significance in ages 65–74. Notably, high deprivation remained significant even in older adults (65–74 years: *HR* = 1.32, 95%*CI*: 1.14–1.53) (Table S10).

In the pathological classification analysis, high-deprivation neighborhoods were associated with elevated risks for adenocarcinoma (*HR* = 1.19, 95%*CI*: 1.04–1.35) and large cell carcinoma (*HR* = 1.58, 95%*CI*: 1.24–2.01). Squamous cell carcinoma (*HR* = 1.75, 95%*CI*: 1.43–2.14) and small cell carcinoma (*HR* = 1.65, 95%*CI*: 1.26–2.14) showed stronger associations with both moderate and high-deprivation neighborhoods (Table S11).

In a sensitivity analysis excluding individuals with a previous diagnosis of lung cancer (1990–1996), the results were similar to those observed in the primary analysis (Table S12), indicating that the association between neighborhood deprivation and lung cancer risk among patients with asthma was robust to the exclusion of individuals with a prior lung cancer diagnosis.

In sensitivity analyses stratified by period of asthma diagnosis (1997–2007 and 2008–2018), the associations between neighborhood deprivation and lung cancer outcomes were generally consistent in direction and magnitude across periods to those observed in the primary analyses (Table S13), suggesting that the findings were not materially influenced by calendar period.

In an additional sensitivity analysis treating neighborhood deprivation as a continuous variable, the results were consistent with those of the primary analysis using categorical deprivation measures. Each one-unit increase in neighborhood deprivation was associated with an adjusted *HR* of 1.08 (95% *CI*: 1.06–1.10) for lung cancer risk and 1.11 (95% *CI*: 1.10–1.12) for mortality due to lung cancer among patients with asthma (Table S14).

In sensitivity analyses stratified by educational level, the association between neighborhood deprivation and lung cancer outcomes was generally consistent across educational categories (Table S15). These findings suggest that the observed association was not entirely attributable to differences in individual educational level.

Figure S4 and Figure S5 display the Kaplan-Meier survival curves showing the time to the first lung cancer incidence and mortality event at varying levels of neighborhood deprivation. As neighborhood deprivation increased, the survival probability of patients affected by lung cancer incidence and mortality decreased, indicating poorer prognosis and demonstrating a graded effect.

## Discussion

In this nationwide cohort study, we found that neighborhood deprivation emerged as a robust independent predictor of both lung cancer development and higher mortality rates among patients with asthma, even after adjusting for demographic and comorbidity factors. This aligns with previous findings that socioeconomic deprivation is associated with adverse cancer outcomes, which may be mediated by overlapping exposures such as environmental pollutants, limited healthcare access, and higher smoking rates in deprived areas [27–29]. The stratified analyses by age and pathological subtypes demonstrated that the consistent association between neighborhood deprivation and lung cancer incidence persisted across age groups and pathological subtypes, suggesting consistency in the observed relationships across these subgroups, thus further emphasizing that the impact of community deprivation on lung cancer outcomes is multifaceted.

Of note, the increased risk observed in older adults (e.g., ≥ 75 years) living in high-deprivation areas compared with those living in low-deprivation areas may reflect their accumulated socioeconomic disadvantage throughout their lives, as well as the negative effects of long-term exposure to environmental carcinogens and delayed healthcare services [30–32]. This is consistent with previous evidence showing that socioeconomic deprivation exacerbates health disparities associated with aging, especially in cancer outcomes [33]. Sex-specific patterns further highlight the complex interplay between social determinants and biological susceptibility. While men exhibited higher overall mortality risks, women in deprived neighborhoods faced steeper gradients in both incidence and mortality. This may reflect sex-based differences in smoking behaviors or biological mechanisms such as hormonal modulation of carcinogenesis [28, 34]. Conversely, the protective role of higher education and urban residence highlights the potential of socioeconomic interventions to reduce lung cancer risks, and this protective effect was stronger among women, which suggests that socioeconomic interventions such as improving health literacy and healthcare infrastructure may benefit this group more, thereby alleviating structural barriers to early diagnosis and treatment [9].

The strong associations observed for COPD and smoking likely reflect, at least in part, their close interrelationship, with COPD serving as a marker of cumulative smoking exposure and other shared risk factors for lung cancer. COPD may act as both a comorbidity and a mediator, amplifying inflammation and epithelial damage that predispose to malignant transformation [35], reinforcing the need for integrated respiratory care in asthma management. The pronounced association between deprivation and aggressive histological subtypes, such as squamous cell (*HR* = 1.75) and small cell carcinomas (*HR* = 1.65), reinforces the role of sustained exposure to tobacco smoke and air pollution, factors disproportionately prevalent in deprived neighborhoods, as key drivers of these malignancies [36, 37]. These findings echo studies linking deprived environments to molecular alterations (e.g., TP53 mutations) that favor aggressive tumor biology [38], highlighting the need for targeted screening and prevention strategies in high-risk communities.

Intriguingly, obesity and hypertension were associated with reduced lung cancer risks in adjusted models, which contrasts with conventional paradigms. While survival bias or diagnostic delays in deprived populations could partially explain this phenomenon [39], emerging evidence suggests genetic loci (e.g., rs79297227) or weight-reducing strategies may modulate cancer progression [40, 41]. Whether the protective effect of obesity and hypertension on lung cancer patients with asthma is caused by survival bias, diagnostic differences, or biological mechanisms is still unclear and deserves further research.

## Limitations

Our study has several limitations. First, we lacked data on certain lung cancer risk factors, including air pollution, unmeasured occupational hazards, passive smoking, and, most importantly, detailed individual smoking data. We partially addressed this by adjusting for COPD (Yes or No) as a proxy for smoking history; although the findings remained significant, they were slightly attenuated after these adjustments. However, it should be noted that a clinical diagnosis of smoking and a history of smoking largely overlap, and both may be collinear with COPD. Since both variables were associated with the outcome, we retained both in the model, as COPD may also serve as a proxy for longer-standing smoking. Nevertheless, because no data was available on the amount, duration, or intensity of smoking, the use of these two binary variables (smoking and COPD) may still leave residual confounding by smoking. For example, individuals with higher deprivation may have smoked more heavily. As tobacco smoking prevalence is also higher in deprived neighborhoods [42], smoking remains a potentially important residual confounder. Given that robust control for smoking status remains challenging in large-scale epidemiological studies, future research should focus on smaller samples with more detailed smoking data to explore this potential mechanism. Second, some individuals may have changed their residence or neighborhood socioeconomic status during the study period. However, our analysis accounted for residential mobility and still found that neighborhood poverty was associated with significantly increased lung cancer incidence and mortality in this cohort of patients with asthma. Third, regarding socioeconomic measurement, although we adjusted simultaneously for both individual‑level indicators and neighborhood deprivation, the latter was derived from area‑level aggregated data and may correlate with individual factors. Our stratification analysis by education suggested robustness, yet residual confounding and potential ecological fallacy remain as limitations. Fourth, the estimates of lung cancer incidence and mortality associated with covariates should be interpreted cautiously, as they may reflect confounding distinct from the primary exposure factors [43]. Furthermore, outcome data were obtained from nationwide hospital and cancer registry records, providing objective outcome ascertainment based on standardized diagnostic coding and reducing the likelihood of outcome misclassification.

## Strengths

Nonetheless, the present study also has several important strengths. Our nationwide cohort included practically all patients with asthma aged 25 years and older in Sweden during the study period, which enhances the generalizability of our findings. The unique Swedish personal identification number assigned to each resident of Sweden (pseudonymized to protect individuals’ integrity) enabled nearly complete follow-up of all patients. Furthermore, outcome data were derived from hospital diagnoses rather than self-reports, eliminating recall bias. Another key strength was the use of small geographical units (SAMS, typically comprising 1000–2000 residents) characterized by relatively homogeneous building types. This granular approach further supports the generalizability of our results, as small neighborhood units have been shown to align closely with residents’ own perceptions of their neighborhoods [44]. Collectively, the findings from previous studies and the present research underscore the importance of improving health outcomes in socioeconomically disadvantaged areas, an ongoing priority in Europe [45].

## Conclusions

In conclusion, this study positions neighborhood deprivation as a potentially critical social determinant of lung cancer in patients with asthma. Public health strategies should prioritize smoking cessation programs, improved asthma management, and equitable resource allocation in deprived communities. Sex and subtype-specific interventions, coupled with policies addressing educational disparities, may further reduce the burden of lung cancer in this vulnerable population. Future research should explore and evaluate targeted interventions in high-risk subgroups.

## Supplementary Information

Supplementary Material 1.

## Data Availability

No datasets were generated or analysed during the current study.

## Abbreviations

HRs: Hazard Ratios
CI: Confidence Intervals
COPD: Chronic Obstructive Pulmonary Disease
ICD: International Classification of Diseases
SAMS: Small Area Market Statistics
