# Supplementary material for: Neighborhood deprivation and lung cancer incidence and mortality in patients with asthma: a nationwide cohort study in Sweden, 1997–2018
Source: Respir Res. 2026 Sep 22;27:370. doi: 10.1186/s12931-026-03927-5 (PMC13613788; doi:10.1186/s12931-026-03927-5)
Supplement: Supplementary file 1 [file 12931_2026_3927_MOESM1_ESM.docx]

**Supplementary materials**

Table S1. Study population and neighborhoods distribution

Table S2. The full code of Directed acyclic graph

Table S3.Population and events of lung cancer incidence and mortality due to lung cancer among asthma patients

Table S4. Association between neighborhood deprivation and lung cancer incidence among asthma patients in men

Table S5. Association between neighborhood deprivation and lung cancer incidence among asthma patients in women

Table S6. Association between neighborhood deprivation and lung cancer incidence among asthma patients

Table S7. Association between neighborhood deprivation and mortality due to lung cancer among asthma patients in men

Table S8. Association between neighborhood deprivation and mortality due to lung cancer among asthma patients in women

Table S9. Association between neighborhood deprivation and mortality due to lung cancer among asthma patients

Table S10. Association between neighborhood deprivation and lung cancer incidence among asthma patients stratified by age

Table S11. Association between neighborhood deprivation and subtypes of lung cancer incidence among asthma patients

Table S12. Association between neighborhood deprivation and lung cancer incidence among asthma patients, after excluding lung cancer diagnosed during 1990-1996, N=595,640

Table S13. Association between neighborhood deprivation and lung cancer incidence and mortality due to lung cancer among asthma patients, by different follow-up periods

Table S14. Association between neighborhood deprivation and lung cancer incidence and mortality due to lung cancer among asthma patients, neighborhood deprivation was added as continuously increasing

Table S15. Association between neighborhood deprivation and lung cancer incidence and mortality due to lung cancer among asthma patients, divided by educational level

Figure S1. The flow chart of the study

Figure S2. Map of Sweden

Figure S3. Directed acyclic graph

Figure S4. Kaplan-Meier survival estimates lung cancer incidence among asthma patients

Figure S5. Kaplan-Meier survival estimates mortality due to lung cancer among asthma patients

| Table S1. Study population and neighborhoods distribution | | | | |
| --- | --- | --- | --- | --- |
| Variables | Low-deprivation | Moderate-deprivation | High-deprivation | Total |
| Number of neighborhoods (%) | 1570 (25.7) | 3476 (56.8) | 1071 (17.5) | 6117 |
| Neighborhood derivation index (SES index) | <-1 | -1-1 | >1 |  |
| Study population (%) | 143691 (24.1) | 335439 (56.3) | 117085 (19.6) | 596215 |
| Mean age (*IQR*) | 52.5 (39-65) | 54.9 (40-69) | 53.4 (38-68) | 54.0 (39-68) |
| Mean follow-up (± *SD*) | 8.6 (± 5.0) | 9.0 (± 5.5) | 8.2 (± 5.4) | 8.7 (± 5.4) |
| Lung cancer incidence (%) | 1028 (18.8) | 3175 (58.0) | 1270 (23.2) | 5473 |
| Standardized overall incidence rate* (per 10000 person years) (95% *CI*) | 4.65 (4.38-4.94) | 5.52 (5.33-5.71) | 7.63 (7.22-8.06) | 5.67 (5.52-5.82) |
| Cumulative rate per 100 individuals | 0.72 (0.65-0.78) | 0.95 (0.91-0.98) | 1.08 (1.03-1.14) | 0.92 (0.89-0.94) |
| Mortality due to lung cancer (%) | 773 (17.4) | 2533 (57.1) | 1133 (25.5) | 4439 |
| Standardized overall mortality rate* (per 10000 person years) (95% *CI*) | 3.40 (3.20-3.61) | 4.11 (3.97-4.25) | 6.47 (6.13-6.83) | 4.34 (4.23-4.46) |
| Cumulative rate per 100 individuals | 0.54 (0.47-0.61) | 0.76 (0.72-0.79) | 0.97 (0.91-1.032) | 0.74 (0.72-0.77) |
| *: Adjusted for the European standardized population. *IQR*=Interquartile range; *SD*=Standardized deviation; *CI*=Confidence interval. | | | | |

Table S2. The full code of Directed acyclic graph

dag {

bb="-10.787,-10.72,8.908,10.175"

"Educational level" [pos="-1.450,-5.034"]

"Family income" [pos="-3.752,-4.894"]

"Immigration status" [pos="-1.292,-8.427"]

"Lung cancer" [outcome,pos="4.966,8.093"]

"Neighborhood deprivation" [exposure,pos="-8.802,-9.690"]

"Region of residence" [pos="4.822,-9.644"]

Age [pos="-5.579,-3.069"]

Alcoholism [pos="-7.018,7.765"]

COPD [pos="-8.672,4.934"]

Gender [pos="3.887,-3.139"]

Smoking [pos="-4.270,8.958"]

"Educational level" -> "Family income"

"Educational level" -> "Lung cancer"

"Educational level" -> "Neighborhood deprivation"

"Family income" -> "Lung cancer"

"Family income" -> "Neighborhood deprivation"

"Immigration status" -> "Educational level"

"Immigration status" -> "Family income"

"Immigration status" -> "Lung cancer"

"Immigration status" -> "Neighborhood deprivation"

"Immigration status" -> "Region of residence"

"Neighborhood deprivation" -> "Lung cancer"

"Neighborhood deprivation" -> Alcoholism

"Neighborhood deprivation" -> COPD

"Neighborhood deprivation" -> Smoking

"Region of residence" -> "Lung cancer"

"Region of residence" -> "Neighborhood deprivation"

"Region of residence" -> Alcoholism

"Region of residence" -> COPD

"Region of residence" -> Smoking

Age -> "Lung cancer"

Age -> Alcoholism

Age -> COPD

Age -> Smoking

Alcoholism -> "Lung cancer"

COPD -> "Lung cancer"

Gender -> "Lung cancer"

Gender -> Alcoholism

Gender -> COPD

Gender -> Smoking

Smoking -> "Lung cancer"

Smoking -> Alcoholism

Smoking -> COPD

}

| Table S3. Population of lung cancer incidence and mortality due to lung cancer among asthma patients | | | | | | | | | | | | | | | | |
| --- | --- | --- | --- | --- | --- | --- | --- | --- | --- | --- | --- | --- | --- | --- | --- | --- |
| Variables | Total Population | |  | Lung cancer incidence | |  | Neighborhood deprivation level-specific rate per 1,000 | | |  | Mortality due to lung cancer | |  | Rate per 1000 in neighborhood deprivation level | | |
|  | No. | % |  | No. | % |  | Low | Moderate | High |  | No. | % |  | Low | Moderate | High |
| **Total population** | 596215 |  |  | 5473 |  |  | 7.2 | 9.5 | 10.8 |  | 4439 |  |  | 5.4 | 7.6 | 9.7 |
| **Age (years)** |  |  |  |  |  |  |  |  |  |  |  |  |  |  |  |  |
| 25-34 | 96077 | 16.1 |  | 38 | 0.7 |  | 0.2 | 0.6 | 0.2 |  | 15 | 0.3 |  | 0.0 | 0.3 | 0.0 |
| 35-44 | 109268 | 18.3 |  | 209 | 3.8 |  | 1.3 | 2.2 | 2.0 |  | 109 | 2.5 |  | 0.5 | 1.2 | 1.0 |
| 45-54 | 105235 | 17.7 |  | 728 | 13.3 |  | 4.3 | 7.5 | 8.8 |  | 556 | 12.5 |  | 3.1 | 5.4 | 8.0 |
| 55-64 | 103619 | 17.4 |  | 1633 | 29.8 |  | 12.3 | 15.8 | 20.1 |  | 1155 | 26.0 |  | 8.2 | 11.3 | 14.8 |
| 65-74 | 94191 | 15.8 |  | 1853 | 33.9 |  | 17.2 | 19.1 | 24.7 |  | 1535 | 34.6 |  | 13.1 | 15.5 | 22.7 |
| ≥75 | 87825 | 14.7 |  | 1012 | 18.5 |  | 11.7 | 10.6 | 14.0 |  | 1069 | 24.1 |  | 11.5 | 11.1 | 15.9 |
| **Sex** |  |  |  |  |  |  |  |  |  |  |  |  |  |  |  |  |
| Males | 235734 | 39.5 |  | 2012 | 36.8 |  | 6.6 | 8.6 | 10.6 |  | 1673 | 37.7 |  | 5.1 | 7.1 | 9.7 |
| Females | 360481 | 60.5 |  | 3461 | 63.2 |  | 7.5 | 10.0 | 11.0 |  | 2766 | 62.3 |  | 5.6 | 7.9 | 9.7 |
| **Educational level** |  |  |  |  |  |  |  |  |  |  |  |  |  |  |  |  |
| Low | 160654 | 26.9 |  | 2392 | 43.7 |  | 14.8 | 14.6 | 15.6 |  | 2163 | 48.7 |  | 11.5 | 13.2 | 15.1 |
| Middle | 163166 | 27.4 |  | 1778 | 32.5 |  | 9.6 | 11.1 | 11.8 |  | 1343 | 30.3 |  | 7.5 | 8.0 | 9.7 |
| High | 272395 | 45.7 |  | 1303 | 23.8 |  | 4.3 | 5.0 | 5.1 |  | 933 | 21.0 |  | 3.0 | 3.5 | 3.9 |
| **Family income** |  |  |  |  |  |  |  |  |  |  |  |  |  |  |  |  |
| Low | 148903 | 25.0 |  | 1445 | 26.4 |  | 7.8 | 9.4 | 11.2 |  | 1368 | 30.8 |  | 7.3 | 8.7 | 11.2 |
| Middle low | 149160 | 25.0 |  | 1748 | 31.9 |  | 8.2 | 12.4 | 13.3 |  | 1518 | 34.2 |  | 7.5 | 10.3 | 12.3 |
| Middle high | 149000 | 25.0 |  | 1421 | 26.0 |  | 8.4 | 9.8 | 10.5 |  | 1064 | 24.0 |  | 5.9 | 7.4 | 8.2 |
| High | 149152 | 25.0 |  | 859 | 15.7 |  | 5.4 | 6.0 | 5.9 |  | 489 | 11.0 |  | 3.1 | 3.5 | 3.0 |
| **Immigrant status** |  |  |  |  |  |  |  |  |  |  |  |  |  |  |  |  |
| Sweden | 495197 | 83.1 |  | 4565 | 83.4 |  | 7.2 | 9.4 | 11.9 |  | 3688 | 83.1 |  | 5.4 | 7.5 | 10.7 |
| Other countries | 101018 | 16.9 |  | 908 | 16.6 |  | 6.8 | 9.9 | 9.0 |  | 751 | 16.9 |  | 5.0 | 8.0 | 7.9 |
| **Region of residence** |  |  |  |  |  |  |  |  |  |  |  |  |  |  |  |  |
| Large cities | 338510 | 56.8 |  | 3287 | 60.1 |  | 7.3 | 10.2 | 11.8 |  | 2618 | 59.0 |  | 5.5 | 8.0 | 10.2 |
| Southern Sweden | 154522 | 25.9 |  | 1390 | 25.4 |  | 7.1 | 9.2 | 10.2 |  | 1189 | 26.8 |  | 5.1 | 7.7 | 10.0 |
| Northern Sweden | 103183 | 17.3 |  | 796 | 14.5 |  | 6.6 | 8.1 | 7.4 |  | 632 | 14.2 |  | 5.2 | 6.3 | 6.7 |
| **Comorbidities** |  |  |  |  |  |  |  |  |  |  |  |  |  |  |  |  |
| COPD | 268289 | 45.0 |  | 3789 | 69.2 |  | 10.7 | 14.5 | 17.1 |  | 2992 | 67.4 |  | 7.8 | 11.2 | 15.1 |
| Alcoholism | 23347 | 3.9 |  | 271 | 5.0 |  | 10.0 | 11.6 | 12.8 |  | 207 | 4.7 |  | 6.9 | 8.8 | 10.5 |
| Smoking | 9495 | 1.6 |  | 283 | 5.2 |  | 34.2 | 30.1 | 27.3 |  | 183 | 4.1 |  | 19.6 | 19.0 | 19.7 |
| COPD: Chronic obstructive pulmonary disease. | | | | | | | | | | | | | | | | |

| Table S4. Association between neighborhood deprivation and lung cancer incidence among asthma patients in men | | | | | | | | |
| --- | --- | --- | --- | --- | --- | --- | --- | --- |
| Variables | Model 1 | |  | Model 2 | |  | Model 3 | |
|  | *HR* | 95% *CI* |  | *HR* | 95% *CI* |  | *HR* | 95% *CI* |
| Neighborhood deprivation (ref. Low) |  |  |  |  |  |  |  |  |
| Moderate | 1.09 | 0.97-1.22 |  | 1.04 | 0.92-1.17 |  | 1.05 | 0.93-1.18 |
| High | 1.61 | 1.41-1.84 |  | 1.36 | 1.19-1.57 |  | 1.37 | 1.19-1.57 |
| Age (years) | 1.06 | 1.05-1.06 |  | 1.06 | 1.05-1.06 |  | 1.06 | 1.06-1.06 |
| Educational level (ref. High) |  |  |  |  |  |  |  |  |
| Low |  |  |  | 1.74 | 1.56-1.93 |  | 1.57 | 1.42-1.76 |
| Middle |  |  |  | 1.55 | 1.38-1.75 |  | 1.43 | 1.27-1.62 |
| Marital status (ref. Married) |  |  |  | 0.94 | 0.85-1.03 |  | 0.92 | 0.84-1.01 |
| Born other countries (ref. Born in Sweden) |  |  |  | 1.56 | 1.38-1.75 |  | 1.50 | 1.34-1.69 |
| Region of residence (ref. Large cities) |  |  |  |  |  |  |  |  |
| Southern Sweden |  |  |  | 0.77 | 0.68-0.84 |  | 0.73 | 0.65-0.81 |
| Northern Sweden |  |  |  | 0.76 | 0.67-0.86 |  | 0.80 | 0.70-0.91 |
| Comorbidities (ref. Non) |  |  |  |  |  |  |  |  |
| COPD |  |  |  |  |  |  | 2.13 | 1.94-2.34 |
| Alcoholism |  |  |  |  |  |  | 1.36 | 1.13-1.64 |
| Smoking |  |  |  |  |  |  | 3.09 | 2.47-3.85 |
| *HR*: Hazards ratio; *CI*: Confidence interval; COPD: Chronic obstructive pulmonary disease. Model 1: Adjusted for age; Model 2: Adjusted for age, educational level, marital status, immigrant status, and region of residence; Model 3: Adjusted for age, educational level, marital status, immigrant status, region of residence, and comorbidities. | | | | | | | | |

| Table S5. Association between neighborhood deprivation and lung cancer incidence among asthma patients in women | | | | | | | | |
| --- | --- | --- | --- | --- | --- | --- | --- | --- |
| Variables | Model 1 | |  | Model 2 | |  | Model 3 | |
|  | *HR* | 95% *CI* |  | *HR* | 95% *CI* |  | *HR* | 95% *CI* |
| Neighborhood deprivation (ref. Low) |  |  |  |  |  |  |  |  |
| Moderate | 1.18 | 1.08-1.29 |  | 1.12 | 1.02-1.22 |  | 1.12 | 1.02-1.22 |
| High | 1.47 | 1.33-1.64 |  | 1.34 | 1.20-1.49 |  | 1.32 | 1.18-1.47 |
| Age (years) | 1.04 | 1.04-1.04 |  | 1.04 | 1.03-1.04 |  | 1.04 | 1.04-1.04 |
| Educational level (ref. High) |  |  |  |  |  |  |  |  |
| Low |  |  |  | 2.06 | 1.87-2.26 |  | 1.83 | 1.67-2.01 |
| Middle |  |  |  | 2.16 | 1.97-2.37 |  | 1.94 | 1.77-2.13 |
| Marital status (ref. Married) |  |  |  | 0.94 | 0.88-1.01 |  | 0.91 | 0.85-0.98 |
| Born other countries (ref. Born in Sweden) |  |  |  | 0.94 | 0.85-1.03 |  | 0.93 | 0.85-1.03 |
| Region of residence (ref. Large cities) |  |  |  |  |  |  |  |  |
| Southern Sweden |  |  |  | 0.81 | 0.75-0.87 |  | 0.75 | 0.69-0.82 |
| Northern Sweden |  |  |  | 0.72 | 0.65-0.80 |  | 0.75 | 0.68-0.83 |
| Comorbidities (ref. Non) |  |  |  |  |  |  |  |  |
| COPD |  |  |  |  |  |  | 2.32 | 2.16-2.50 |
| Alcoholism |  |  |  |  |  |  | 1.49 | 1.25-1.76 |
| Smoking |  |  |  |  |  |  | 2.97 | 2.56-3.44 |
| *HR*: Hazards ratio; *CI*: Confidence interval; COPD: Chronic obstructive pulmonary disease. Model 1: Adjusted for age; Model 2: Adjusted for age, educational level, marital status, immigrant status, and region of residence; Model 3: Adjusted for age, educational level, marital status, immigrant status, region of residence, and comorbidities. | | | | | | | | |

| Table S6. Association between neighborhood deprivation and lung cancer incidence among asthma patients | | | | | | | | |
| --- | --- | --- | --- | --- | --- | --- | --- | --- |
| Variables | Model 1 | |  | Model 2 | |  | Model 3 | |
|  | *HR* | 95% *CI* |  | *HR* | 95% *CI* |  | *HR* | 95% *CI* |
| Neighborhood deprivation (ref. Low) |  |  |  |  |  |  |  |  |
| Moderate | 1.15 | 1.07-1.23 |  | 1.09 | 1.02-1.17 |  | 1.10 | 1.02-1.18 |
| High | 1.52 | 1.40-1.65 |  | 1.36 | 1.25-1.48 |  | 1.35 | 1.24-1.47 |
| Age (years) | 1.05 | 1.04-1.05 |  | 1.04 | 1.04-1.05 |  | 1.05 | 1.04-1.05 |
| Sex to males (ref. Females) |  |  |  | 0.99 | 0.94-1.05 |  | 1.00 | 0.94-1.05 |
| Educational level (ref. High) |  |  |  |  |  |  |  |  |
| Low |  |  |  | 1.87 | 1.74-2.00 |  | 1.68 | 1.56-1.80 |
| Middle |  |  |  | 1.87 | 1.74-2.01 |  | 1.71 | 1.59-1.83 |
| Marital status (ref. Married) |  |  |  | 0.90 | 0.85-0.95 |  | 0.88 | 0.83-0.93 |
| Born other countries (ref. Born in Sweden) |  |  |  | 1.13 | 1.04-1.21 |  | 1.10 | 1.03-1.19 |
| Region of residence (ref. Large cities) |  |  |  |  |  |  |  |  |
| Southern Sweden |  |  |  | 0.79 | 0.74-0.84 |  | 0.74 | 0.70-0.79 |
| Northern Sweden |  |  |  | 0.73 | 0.68-0.79 |  | 0.77 | 0.71-0.83 |
| Comorbidities (ref. Non) |  |  |  |  |  |  |  |  |
| COPD |  |  |  |  |  |  | 2.26 | 2.13-2.39 |
| Alcoholism |  |  |  |  |  |  | 1.41 | 1.24-1.60 |
| Smoking |  |  |  |  |  |  | 3.09 | 2.74-3.50 |
| *HR*: Hazards ratio; *CI*: Confidence interval; COPD: Chronic obstructive pulmonary disease. Model 1: Adjusted for age; Model 2: Adjusted for age, sex, educational level, marital status, immigrant status, and region of residence; Model 3: Adjusted for age, sex, educational level, marital status, immigrant status, region of residence, and comorbidities. | | | | | | | | |

| Table S7. Association between neighborhood deprivation and mortality due to lung cancer among asthma patients in men | | | | | | | | |
| --- | --- | --- | --- | --- | --- | --- | --- | --- |
| Variables | Model 1 | |  | Model 2 | |  | Model 3 | |
|  | *HR* | 95% *CI* |  | *HR* | 95% *CI* |  | *HR* | 95% *CI* |
| Neighborhood deprivation (ref. Low) |  |  |  |  |  |  |  |  |
| Moderate | 1.23 | 1.17-1.29 |  | 1.08 | 1.02-1.13 |  | 1.10 | 1.04-1.15 |
| High | 1.64 | 1.55-1.74 |  | 1.38 | 1.30-1.47 |  | 1.40 | 1.32-1.49 |
| Age (years) | 1.09 | 1.09-1.09 |  | 1.09 | 1.09-1.09 |  | 1.09 | 1.09-1.09 |
| Educational level (ref. High) |  |  |  |  |  |  |  |  |
| Low |  |  |  | 2.16 | 2.07-2.26 |  | 2.00 | 1.91-2.09 |
| Middle |  |  |  | 1.65 | 1.56-1.74 |  | 1.53 | 1.45-1.62 |
| Marital status (ref. Married) |  |  |  | 1.11 | 1.07-1.15 |  | 1.10 | 1.06-1.14 |
| Born other countries (ref. Born in Sweden) |  |  |  | 1.15 | 1.08-1.21 |  | 1.12 | 1.06-1.18 |
| Region of residence (ref. Large cities) |  |  |  |  |  |  |  |  |
| Southern Sweden |  |  |  | 0.97 | 0.93-1.01 |  | 0.96 | 0.93-1.00 |
| Northern Sweden |  |  |  | 1.01 | 0.97-1.06 |  | 1.06 | 1.01-1.11 |
| Comorbidities (ref. Non) |  |  |  |  |  |  |  |  |
| COPD |  |  |  |  |  |  | 1.69 | 1.63-1.76 |
| Alcoholism |  |  |  |  |  |  | 1.81 | 1.67-1.95 |
| Smoking |  |  |  |  |  |  | 1.21 | 1.04-1.41 |
| *HR*: Hazards ratio; *CI*: Confidence interval; COPD: Chronic obstructive pulmonary disease. Model 1: Adjusted for age; Model 2: Adjusted for age, educational level, marital status, immigrant status, and region of residence; Model 3: Adjusted for age, educational level, marital status, immigrant status, region of residence, and comorbidities. | | | | | | | | |

| Table S8. Association between neighborhood deprivation and mortality due to lung cancer among asthma patients in women | | | | | | | | |
| --- | --- | --- | --- | --- | --- | --- | --- | --- |
| Variables | Model 1 | |  | Model 2 | |  | Model 3 | |
|  | *HR* | 95% *CI* |  | *HR* | 95% *CI* |  | *HR* | 95% *CI* |
| Neighborhood deprivation (ref. Low) |  |  |  |  |  |  |  |  |
| Moderate | 1.26 | 1.21-1.32 |  | 1.14 | 1.09-1.19 |  | 1.15 | 1.10-1.20 |
| High | 1.66 | 1.58-1.74 |  | 1.44 | 1.38-1.52 |  | 1.46 | 1.39-1.54 |
| Age (years) | 1.10 | 1.09-1.10 |  | 1.09 | 1.09-1.09 |  | 1.10 | 1.09-1.10 |
| Educational level (ref. High) |  |  |  |  |  |  |  |  |
| Low |  |  |  | 2.35 | 2.26-2.45 |  | 2.17 | 2.08-2.26 |
| Middle |  |  |  | 1.81 | 1.73-1.90 |  | 1.68 | 1.60-1.76 |
| Marital status (ref. Married) |  |  |  | 1.12 | 1.08-1.16 |  | 1.11 | 1.07-1.15 |
| Born other countries (ref. Born in Sweden) |  |  |  | 1.03 | 0.98-1.08 |  | 1.01 | 0.96-1.06 |
| Region of residence (ref. Large cities) |  |  |  |  |  |  |  |  |
| Southern Sweden |  |  |  | 1.05 | 1.01-1.09 |  | 1.03 | 0.99-1.07 |
| Northern Sweden |  |  |  | 1.04 | 1.00-1.08 |  | 1.07 | 1.03-1.12 |
| Comorbidities (ref. Non) |  |  |  |  |  |  |  |  |
| COPD |  |  |  |  |  |  | 1.63 | 1.58-1.69 |
| Alcoholism |  |  |  |  |  |  | 1.73 | 1.56-1.93 |
| Smoking |  |  |  |  |  |  | 1.32 | 1.17-1.49 |
| *HR*: Hazards ratio; *CI*: Confidence interval; COPD: Chronic obstructive pulmonary disease. Model 1: Adjusted for age; Model 2: Adjusted for age, educational level, marital status, immigrant status, and region of residence; Model 3: Adjusted for age, educational level, marital status, immigrant status, region of residence, and comorbidities. | | | | | | | | |

| Table S9. Association between neighborhood deprivation and mortality due to lung cancer among asthma patients | | | | | | | | |
| --- | --- | --- | --- | --- | --- | --- | --- | --- |
| Variables | Model 1 | |  | Model 2 | |  | Model 3 | |
|  | *HR* | 95% *CI* |  | *HR* | 95% *CI* |  | *HR* | 95% *CI* |
| Neighborhood deprivation (ref. Low) |  |  |  |  |  |  |  |  |
| Moderate | 1.25 | 1.21-1.29 |  | 1.11 | 1.07-1.15 |  | 1.13 | 1.09-1.16 |
| High | 1.64 | 1.58-1.71 |  | 1.41 | 1.36-1.47 |  | 1.43 | 1.38-1.49 |
| Age (years) | 1.09 | 1.09-1.09 |  | 1.09 | 1.09-1.09 |  | 1.09 | 1.09-1.10 |
| Sex to males (ref. Females) |  |  |  | 1.42 | 1.38-1.45 |  | 1.41 | 1.37-1.44 |
| Educational level (ref. High) |  |  |  |  |  |  |  |  |
| Low |  |  |  | 2.26 | 2.19-2.33 |  | 2.08 | 2.02-2.14 |
| Middle |  |  |  | 1.73 | 1.67-1.79 |  | 1.60 | 1.55-1.66 |
| Marital status (ref. Married) |  |  |  | 1.12 | 1.10-1.15 |  | 1.11 | 1.08-1.13 |
| Born other countries (ref. Born in Sweden) |  |  |  | 1.08 | 1.04-1.11 |  | 1.05 | 1.01-1.09 |
| Region of residence (ref. Large cities) |  |  |  |  |  |  |  |  |
| Southern Sweden |  |  |  | 1.01 | 0.98-1.04 |  | 1.00 | 0.97-1.02 |
| Northern Sweden |  |  |  | 1.03 | 1.00-1.06 |  | 1.07 | 1.03-1.10 |
| Comorbidities (ref. Non) |  |  |  |  |  |  |  |  |
| COPD |  |  |  |  |  |  | 1.67 | 1.63-1.70 |
| Alcoholism |  |  |  |  |  |  | 1.79 | 1.68-1.91 |
| Smoking |  |  |  |  |  |  | 1.27 | 1.16-1.40 |
| *HR*: Hazards ratio; *CI*: Confidence interval; COPD: Chronic obstructive pulmonary disease. Model 1: Adjusted for age; Model 2: Adjusted for age, sex, educational level, marital status, immigrant status, and region of residence; Model 3: Adjusted for age, sex, educational level, marital status, immigrant status, region of residence, and comorbidities. | | | | | | | | |

| Table S10. Association between neighborhood deprivation and lung cancer incidence among asthma patients stratified by age | | | | | | | |
| --- | --- | --- | --- | --- | --- | --- | --- |
| Age (years) | Low |  | Moderate | |  | High | |
|  | Ref. |  | *HR** | 95% *CI* |  | *HR** | 95% *CI* |
| <55 | 1.00 |  | 1.37 | 1.14-1.63 |  | 1.43 | 1.16-1.77 |
| 55-64 | 1.00 |  | 1.13 | 0.99-1.29 |  | 1.44 | 1.23-1.69 |
| 65-74 | 1.00 |  | 1.05 | 0.93-1.19 |  | 1.32 | 1.14-1.53 |
| ≥ 75 | 1.00 |  | 0.96 | 0.82-1.14 |  | 1.39 | 1.15-1.69 |
| *: Adjusted for age, sex, educational level, marital status, immigrant status, region of residence, and comorbidities. *HR*: Hazards ratio; *CI*: Confidence interval. | | | | | | | |

| Table S11. Association between neighborhood deprivation and subtypes of lung cancer incidence among asthma patients | | | | | | | | |  |
| --- | --- | --- | --- | --- | --- | --- | --- | --- | --- |
| Subtypes | Low |  | Moderate | |  | High | | |  |
|  | Ref. |  | *HR** | 95% *CI* |  | *HR** | 95% *CI* | |  |
| Adenocarcinoma (N=2281, 41.7%) | 1.00 |  | 1.01 | 0.91-1.12 |  | 1.19 | | 1.04-1.35 | |
| Squamous cell carcinoma (N=1085, 19.8%) | 1.00 |  | 1.37 | 1.15-1.64 |  | 1.75 | | 1.43-2.14 | |
| Small cell carcinoma (N=613, 11.2%) | 1.00 |  | 1.34 | 1.07-1.68 |  | 1.65 | | 1.26-2.14 | |
| Large cell carcinoma (N=677, 12.4%) | 1.00 |  | 1.11 | 0.90-1.37 |  | 1.58 | | 1.24-2.01 | |
| *: Adjusted for age, sex, educational level, marital status, immigrant status, region of residence, and comorbidities. *HR*: Hazards ratio; *CI*: Confidence interval. | | | | | | | | |  |

| Table S12. Association between neighborhood deprivation and lung cancer incidence among asthma patients, after excluding lung cancer diagnosed during 1990-1996, N=595,640 | | |
| --- | --- | --- |
| Variables | *HR** | 95% *CI* |
| Neighborhood deprivation (ref. Low) |  |  |
| Moderate | 1.10 | 1.02-1.18 |
| High | 1.35 | 1.24-1.47 |
| *: Adjusted for age, sex, educational level, marital status, immigrant status, region of residence, and comorbidities. *HR*: Hazards ratio; *CI*: Confidence interval. | | |

| Table S13. Association between neighborhood deprivation and lung cancer incidence and mortality due to lung cancer among asthma patients, by different follow-up periods | | | | | | | |
| --- | --- | --- | --- | --- | --- | --- | --- |
| Variables | Asthma diagnosed 1997-2007, N=201,939 | | |  | Asthma diagnosed 2008-2018, N=339,341 | | |
|  | Events | *HR** | 95% *CI* |  | Events | *HR** | 95% *CI* |
| **Lung cancer incidencce** |  |  |  |  |  |  |  |
| Neighborhood deprivation (ref. Low) | 476 | 1.00 |  |  | 424 | 1.00 |  |
| Moderate | 1543 | 1.14 | 1.02-1.26 |  | 1127 | 1.14 | 1.01-1.27 |
| High | 551 | 1.35 | 1.18-1.52 |  | 565 | 1.49 | 1.30-1.69 |
| **Mortality due to lung cancer** |  |  |  |  |  |  |  |
| Neighborhood deprivation (ref. Low) | 375 | 1.00 |  |  | 270 | 1.00 |  |
| Moderate | 1269 | 1.16 | 1.10-1.21 |  | 753 | 1.18 | 1.11-1.26 |
| High | 499 | 1.41 | 1.34-1.49 |  | 453 | 1.99 | 1.86-2.14 |
| *: Adjusted for age, sex, educational level, marital status, immigrant status, region of residence, and comorbidities. *HR*: Hazards ratio; *CI*: Confidence interval. | | | | | | | |

| Table S14 Association between neighborhood deprivation and lung cancer incidence and mortality due to lung cancer among asthma patients, neighborhood deprivation was added as continuously increasing | | | | | |
| --- | --- | --- | --- | --- | --- |
| Variables | Lung cancer incidence | |  | Mortality due to lung cancer | |
|  | *HR** | 95% *CI* |  | *HR** | 95% *CI* |
| Neighborhood deprivation (continuously increasing) | 1.08 | 1.06-1.10 |  | 1.11 | 1.10-1.12 |
| Age (years) | 1.05 | 1.04-1.05 |  | 1.09 | 1.09-1.10 |
| Sex to males (ref. Females) | 1.00 | 0.94-1.05 |  | 1.40 | 1.37-1.44 |
| Educational level (ref. High) |  |  |  |  |  |
| Low | 1.66 | 1.55-1.79 |  | 2.06 | 2.00-2.12 |
| Middle | 1.70 | 1.58-1.83 |  | 1.60 | 1.55-1.66 |
| Marital status (ref. Married) | 0.88 | 0.83-0.93 |  | 1.10 | 1.08-1.13 |
| Born other countries (ref. Born in Sweden) | 1.08 | 1.00-1.16 |  | 1.01 | 0.98-1.05 |
| Region of residence (ref. Large cities) |  |  |  |  |  |
| Southern Sweden | 0.74 | 0.70-0.79 |  | 1.00 | 0.97-1.02 |
| Northern Sweden | 0.77 | 0.72-0.84 |  | 1.08 | 1.04-1.11 |
| Comorbidities (ref. Non) |  |  |  |  |  |
| COPD | 2.26 | 2.13-2.39 |  | 1.67 | 1.63-1.71 |
| Alcoholism | 1.41 | 1.24-1.59 |  | 1.77 | 1.67-1.89 |
| Smoking | 3.07 | 2.72-3.47 |  | 1.26 | 1.14-1.38 |
| *: Adjusted for age, sex, educational level, marital status, immigrant status, region of residence, and comorbidities. *HR*: Hazards ratio; *CI*: Confidence interval; COPD: Chronic obstructive pulmonary disease. | | | | | |

| Table S15. Association between neighborhood deprivation and lung cancer incidence and mortality due to lung cancer among asthma patients, divided by educational level | | | | | | | | |
| --- | --- | --- | --- | --- | --- | --- | --- | --- |
| Variables | Educational level | | | | | | | |
|  | Low | |  | Middle | |  | High | |
|  | *HR** | 95% *CI* |  | *HR** | 95% *CI* |  | *HR** | 95% *CI* |
| **Lung cancer incidence** |  |  |  |  |  |  |  |  |
| Neighborhood deprivation (ref. Low) | 1.00 |  |  | 1.00 |  |  | 1.00 |  |
| Moderate | 1.01 | 0.89-1.14 |  | 1.17 | 1.03-1.33 |  | 1.14 | 1.00-1.29 |
| High | 1.32 | 1.16-1.51 |  | 1.37 | 1.18-1.59 |  | 1.20 | 1.01-1.43 |
| **Mortality due to lung cancer** |  |  |  |  |  |  |  |  |
| Neighborhood deprivation (ref. Low) | 1.00 |  |  | 1.00 |  |  | 1.00 |  |
| Moderate | 1.13 | 1.08-1.18 |  | 1.14 | 1.07-1.22 |  | 1.15 | 1.08-1.23 |
| High | 1.50 | 1.42-1.58 |  | 1.38 | 1.28-1.49 |  | 1.31 | 1.21-1.42 |
| *: Adjusted for age, sex, marital status, immigrant status, region of residence, and comorbidities. *HR*: Hazards ratio; *CI*: Confidence interval. | | | | | | | | |

**Figure S1.** The flow chart of the study

**Figure S2.** Map of Sweden (The map was adapted from Statistic Sweden: Cancer in incidence Sweden 2006)

Region of residence was divided into three groups: Large cities ('01','12', '14'), Southern Sweden ('03','04','05','06','07','08','09','10','11', '13','15','16','18','19','21'), and Northern Sweden (’20’, 22’,’23’,’24’,’25’)

**Figure S3.** Directed acyclic graph.

This model was constructed at [www.dagitty.net](http://www.dagitty.net) (v3.0) to obtain a minimal sufficient adjustment set for the multiple regression model. Yellow circle for exposure, blue circle for outcome, pink circle for ancestor of exposure and outcome, pink arrow for biasing path, and green arrow for causal path.

**Figure S4.** Kaplan-Meier survival estimates lung cancer incidence among asthma patients

**Figure S5.** Kaplan-Meier survival estimates mortality due to lung cancer among asthma

patients.
